# Solution Processable CrN Thin Films: Thickness-Dependent Electrical Transport Properties

**DOI:** 10.3390/ma13020417

**Published:** 2020-01-16

**Authors:** Zhenzhen Hui, Xuzhong Zuo, Longqiang Ye, Xuchun Wang, Xuebin Zhu

**Affiliations:** 1 College of Chemistry and Materials Engineering, Anhui Science and Technology University, Fengyang 233100, China; yelq@ahstu.edu.cn (L.Y.); xuchun.wang@163.com (X.W.); 2 College of Electrical and Electronic Engineering, Anhui Science and Technology University, Fengyang 233100, China; zxz1003@mail.ustc.edu.cn; 3 Key Laboratory of Materials Physics, Institute of Solid State Physics, University of Chinese Academy of Sciences, Hefei 230031, China; xbzhu@issp.ac.cn

**Keywords:** chromium nitride, thin films, thickness-dependent, electrical transport properties, chemical solution deposition

## Abstract

Thickness is a very important parameter with which to control the microstructures, along with physical properties in transition-metal nitride thin films. In work presented here, CrN films with different thicknesses (from 26 to 130 nm) were grown by chemical solution deposition. The films are pure phase and polycrystalline. Thickness dependence of microstructures and electrical transport behavior were studied. With the increase of films thickness, grain size and nitrogen content are increased, while resistivity, zero-field sensitivity and magnetoresistance are decreased. In the temperature range of 5–350 K, all samples exhibited semiconductor-like properties with d*ρ*/d*T* < 0. For the range above and below the Néel temperature, the resistivity can be fitted by the thermal activation model and the two-dimensional weak localization (2D-WL) model, respectively. The ultra-low magnetoresistance at a low temperature under high magnetic fields with a large zero-field sensitivity was observed in the CrN thin films. The zero-field sensitivity can be effectively tuned to 10^−2^ K^−1^ at 5 K with a magnetoresistance of less than 1% at 2 K under 14 T by reasonably controlling the thickness.

## 1. Introduction

As a typical of transition-metal nitride, chromium nitride (CrN) with an NaCl structure has been widely investigated in the past few years. Its striking performance in applications including electronic devices is thought to be on account of the magnetic ordering; surface protective coatings, due to the good friction resistance; and cutting tools due to the high hardness [1,2,3,4,5,6]. Compared with the magnetic transition from antiferromagnetism to paramagnetism reported extensively in the literature, the electrical transport behavior of CrN thin films exhibits large variations. The transport properties transform from a semiconductor-like behavior to a metallic behavior, coupled with the tremendous change of resistance value at 300 K. For example, some studies report that the room temperature resistivity of CrN thin films varies from 0.1 to 2 mΩ·cm, and the electrical transport behavior is characterized by metallic conductivity. Others report that the transport properties of CrN thin films are similar to those of the semiconductor, and the room temperature resistivity is 10^3^ mΩ·cm [6,7,8]. The difference from the electrical transport properties is usually attributed to the change of stoichiometric ratio and grain size of samples. On the other hand, CrN with low magnetoresistance has attracted much interest in applications of temperature sensors under high magnetic fields. Accurate temperature measurement is required for many cryogenic sensors. However, it is always as a curse due to the obvious magnetoresistance under high magnetic fields [9]. Currently, the main components of the most commercially adapted bulks are carbon, carbon-glass and carbon ceramic, and the thin films are based on ruthenium oxide and ceramic nitride oxide (CERNOX), which show typical magnetic-field-induced resistance errors of 1.5% at 4.2 K under 15 T [10]. Improvement of the measurement accuracy as well as providing an alternative with low-cost are very desirable for the cryogenic sensors under high magnetic fields. To search for new types of the sensors, two important parameters—including the zero-field sensitivity (*S* = |(d*ρ*/d*T*)/*ρ*|, where *ρ* is resistivity and *T* is the measured temperature) and the magnetoresistance (*MR* = (*ρ_H_* − *ρ_0_*)/ *ρ_0_* × 100%, where *ρ_H_* and *ρ_0_* are the resistivity in the presence and absence of an external magnetic field, respectively), should be considered [11,12]. The *S* should be as large as possible, while for the *MR* the value should be as low as possible. It has been recognized that thin films with semiconductor-like transport behavior will give rise to a large *S* value. Moreover, it is observed that transition-metal nitrides such as CERNOX have relatively low MR values [11,12,13]. To optimize these parameters, it is conjectured that nanostructured CrN thin films will be ideal candidates for cryogenic sensors under high magnetic fields, since the transport properties can be easily tuned by controlling the thickness, resulting in relatively large zero-field sensitivity. Moreover, through the stoichiometry adjustment, the magnetoresistance can be kept to a relatively low value. At present, the CrN thin films have been successfully fabricated by RF reactive magnetron sputtering, molecular beam epitaxy, pulsed laser deposition, ion beam assisted deposition and so on [7,8,14,15,16,17]. As an alternative approach, chemical solution deposition (CSD) can make the precursor film mix at the atomic level. The obtained film was stoichiometric with a large area [18,19]. And this method has been successfully applied in our previous reports [20,21].

In our study, nanocrystalline CrN thin films with different thickness were prepared by CSD, which provides a good stoichiometric control and chemical uniformity for the preparation of nitride thin films. The thickness dependence on the microstructure, nitrogen content, zero-field sensitivity and magnetoresistance, along with electrical transport properties were systematically studied.

## 2. Materials and Methods

**Figure 1:**
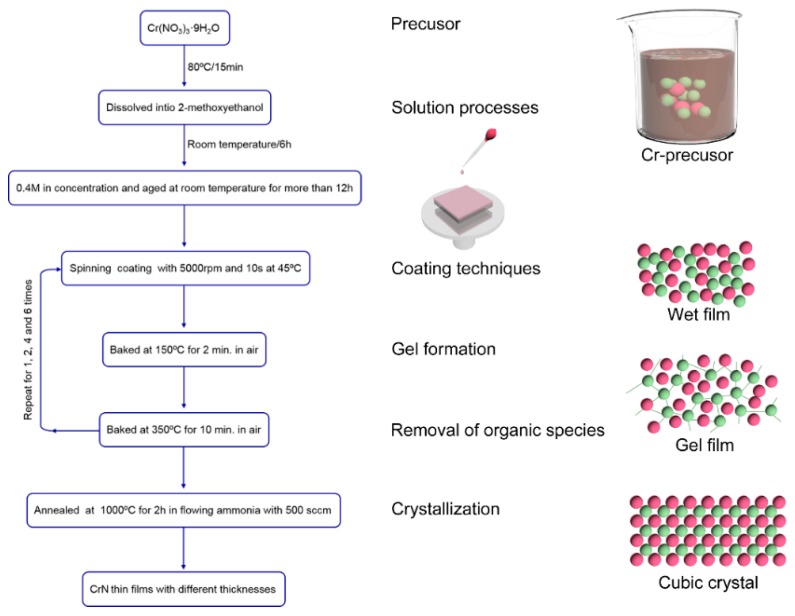
Flow chart of preparation of CrN thin films by the CSD method.

Chromium nitrate (Cr(NO_3_)_3_·9H_2_O), due to sufficient solubility, was used as the precursor material. It was dissolved into 2-methoxyethanol (2-MOE) to prepare the coating solution. The concentration of that solution was 0.4 mol/L. Then, the solution was allowed to stand for 12 h while waiting for deposition. The coating technique used in this study was spin-coating deposition. The substrates used for depositing the CrN films were SrTiO_3_ (100) single crystal substrates. Prior to deposition of the films, to increase the cleanliness and acceptable wetting of the substrate, all substrates were ultrasonically cleaned in acetone, absolute ethanol and distilled water for 5 min in sequence, and finally washed in a plasma cleaner for 10 min. After the completion of the preparation, the thin films were deposited by the spin-coating method. The deposition temperature was 45 °C with the rotation speed of 5000 rpm and the deposition time of 10 s. Then, the gel film was baked at 150 °C for 2 min, and pyrolyzed for 10 min in the air at 350 °C. The term pyrolysis is predominantly used to describe the decomposition of the organic matrix in air. In order to obtain thin films with different thicknesses, the above process was repeated 1, 2, 4 and 6 times. Finally, all the derived thin films were annealed at 1000 °C for 2 h in ammonia atmosphere. The post-annealing treatment may be employed to initiate crystallization, to improve microstructure and to increase film density. The thin film thicknesses of spin-coating 1, 2, 4 and 6 times were 26, 44, 82 and 130 nm, respectively. For convenience of description, the thin films we obtained are abbreviated as T_26_, T_44_, T_82_ and T_130_. The processing flow chart is shown in the Figure 1.

The phase composition and quality of the CrN thin films were analyzed by small angle X-ray diffraction at room temperature using monochrome Cu-K_α_ ray (SAXRD, X’Pert PRO, PANalytical, Almelo, The Netherlands). The surface morphology and thickness of each film were measured by field emission scanning electron microscopy (FE-SEM, Sirion 200, FEI, Hillsboro, OR, USA). The microstructures were further analyzed by high-resolution transmission electron microscopy (HRTEM, JEM-2010, JEOL Ltd., Tokyo, Japan). The chemical states of chromium and nitrogen were analyzed by X-ray photoelectron spectroscopy (XPS, ESCALAB250, Thermo, Waltham, MA, USA). The electrical transport properties and Hall measurements at room temperature were investigated on the physical properties measurement system (PPMS, Quantum Design, San Diego, CA, USA).

## 3. Results and Discussion

### 3.1. Structural and Surface Morphology Studies

**Figure 2:**
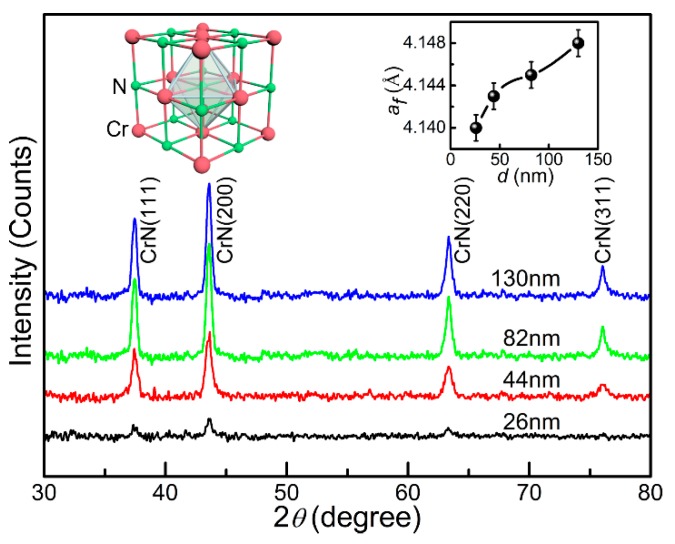
Grazing incidence XRD of CrN thin films with different thicknesses. The crystal structure of CrN and the thickness dependence of lattice constants are shown in the corresponding inset.

Figure 2 shows the small angle X-ray diffraction patterns of CrN thin films with different thicknesses at room temperature. All the films are single-phase and have no detectable impurities. The X-ray diffraction patterns can be indexed by the space group of Fm-3m (PDF card number 03-065-9001) with a rock-soft structure, as shown in the left inset of Figure 2, which is consistent with the previous results [22,23]. The derived thin films were randomly oriented and had a polycrystalline structure. The polycrystalline film was due to the homogeneous nucleation in the bulk thin film of CrN. Theoretically, a large thermal expansion coefficient difference and lattice mismatch increase the interfacial energy between the CrN thin film and the SrTiO_3_ (001) substrate, which leads to the homogeneous nucleation in the most bulk thin film, resulting in the formation of polycrystalline CrN thin films [24,25]. The degree of lattice mismatch between the CrN thin film and the substrate was obtained by the formula *ε* = (*a_f_* − *a_s_*)/*a_f_* × 100%, where *a_f_* and *a_s_* represent the lattice constant of the CrN thin film (4.140~4.148 Å) and the substrate (3.90 Å), respectively. With the increase of thin film thickness, the crystalline quality was improved gradually. The lattice constant of any CrN thin film was calculated by Bragg formula (2*d*sin*θ* = n*λ*) and the formula between the interplanar spacing *d* and the lattice constant *a* of the cubic system *d* = *a*/(*h*^2^ + *k*^2^ + *l*^2^)^1/2^ (*h*, *k* and *l* are the Miller indices), which uses the positions of CrN (111), CrN (200), CrN (220) and CrN (311) peaks. The final lattice constant’s value is the average value of the four abovementioned results. By analogy, the lattice constants of the other three samples can be obtained. For the derived CrN thin films, the lattice constants were calculated as 4.140, 4.143, 4.145 and 4.148 Å for the T_26_, the T_44_, the T_82_ and the T_130_, respectively. The variation of lattice constants for the CrN thin films with different thicknesses as due to the different nitrogen content in each system, as shown in the right inset of Figure 2. According to the literature, the more the nitrogen content, the larger the lattice constant [26]. In addition, for the T_130_ thin film, the lattice constant is 4.148 Å, which is consistent with the lattice constant of 4.149 Å for the CrN thin film with a stoichiometric ratio reported previously [26]. It was proven that the chemical composition of the T_130_ thin film reached near stoichiometry. With decreasing thickness, the lattice constant is gradually decreased.

**Figure 3:**
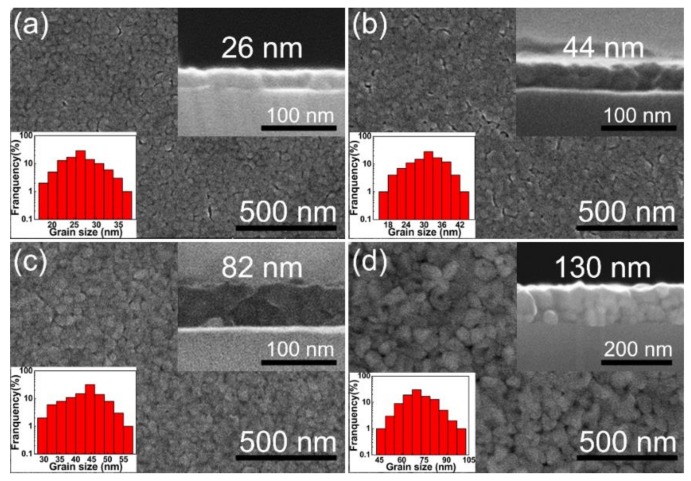
FE-SEM results of the CrN thin films with different thicknesses: (**a**) 26, (**b**) 44, (**c**) 82 and (**d**) 130 nm. The thicknesses and the histograms of the grain size are shown in the corresponding insets.

The results of field emission scanning electron microscopy (FE-SEM) for the surface morphologies of all the films are shown in Figure 3a–d. The resulting CrN thin films had a relatively smooth, uniform, and dense surface. Since the grain size plays an important role in both the microstructures and other properties of the CrN films, in order to obtain an average grain size, we measured the sizes of hundreds of grains using an image analyzer. The corresponding FE-SEM results show the histogram of grain size distribution of the derived CrN films. From the analysis of the figure, the average grain sizes were 26, 31, 44 and 73 nm for the T_26_, the T_44_, the T_82_ and the T_130_, respectively. According to the specimen thickness effect, the in-plane grain growth size is less than or equal to the thickness in a polycrystalline thin film. In the thinner CrN films, because of the smaller grain size and more grain boundaries, atoms diffusion along and across grain boundaries will be more hindered during the process of grain growth. Therefore, the thinner the thin films, the more difficult the three-dimensional diffusion is. The results show that the average grain size increases with increasing thickness, which may be due to the enhancement of atomic diffusion during grain growth and the successive pyrolysis steps during the preparing process [25,27,28]. The thicknesses of the obtained CrN films were found to be 26, 44, 82 and 130 nm for the T_26_, the T_44_, the T_82_ and the T_130_, respectively, as shown in the corresponding cross-sectional SEM image. It is indicated that the thickness was 20–25 nm for each coating layer.

**Figure 4:**
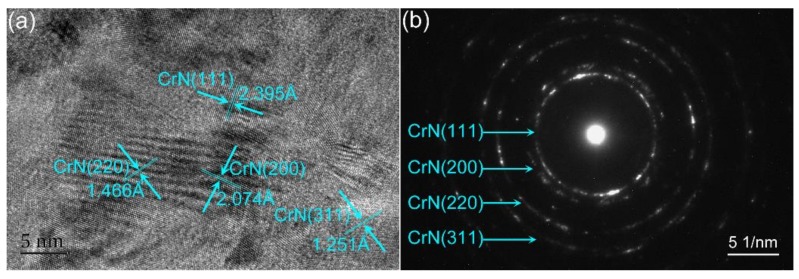
(**a**) Surface HRTEM result of the T130 thin film. (**b**) The corresponding SAED pattern of surface lattice stripes.

In order to further investigate the microstructures of the CrN thin films, TEM measurements of the typical sample T_130_ were carried out, and the results are shown in Figure 4. From the surface high-resolution TEM (HRTEM) image, as shown in Figure 4a, randomly oriented grains with blurred grain boundaries can be observed, which indicates that the derived CrN thin film is polycrystalline, and the result is the same as from the XRD measurements. The *d* spacing, as indexed, can be attributed to CrN (111), CrN (200), CrN (220) and CrN (311) planes. The corresponding selected-area electron diffraction (SAED) pattern is presented as diffraction rings, as shown in the Figure 4b. The diffraction rings from center to edge are indexed as (111), (200), (220) and (311) respectively, indicating the polycrystalline characteristics. The crystal structure of the derived CrN thin film can be indexed as a rock-soft cubic structure with the space group of Fm-3m (PDF card number 03-065-9001), which is the same as in previous reports [22,23].

### 3.2. Nitrogen Content Variations

**Figure 5:**
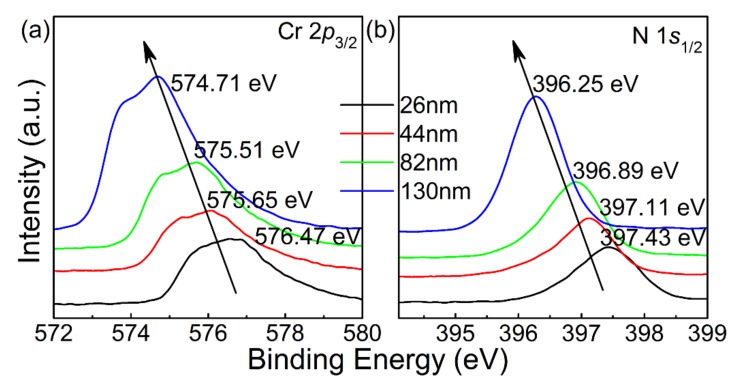
XPS spectra of the CrN thin films with different thicknesses: (**a**) Cr 2p and (**b**) N 1s.

To investigate the variation of nitrogen content and the stoichiometry of all the derived CrN thin films, X-ray photoelectron spectroscopy (XPS) of Cr 2*p* and N 1*s* is shown in Figure 5. The spectra were calibrated using C1s core level peak located at 284.8 eV. It is seen that the peak value centered at ≈575 eV can be attributed to the Cr 2*p*_3/2_. In addition, due to the Doniach-sunjic effect [29], the shape of the Cr 2*p* peaks is asymmetric. As mentioned above, that is usually observed in the XPS spectra of transition metals. In Figure 5b, the peak value centered at ≈396 eV can be attributed to the N 1*s*_1/2_, which proves that the nitrogen element exists in the corresponding CrN films [2]. As described in previous reports, the binding energies of Cr 2*p* and N 1*s* decrease as the nitrogen content increases [30,31]. The binding energies of Cr 2*p*_3/2_ and N 1*s*_1/2_ were 576.47 and 397.43 eV for T_26_ 575.65; 397.11 eV for T_44_ 575.51; 396.89 eV for T_82_; and 574.71 and 396.25 eV for T_130_, respectively. As listed in Table 1, it is confirmed that the nitrogen content increases with the decrease of binding energy of the derived CrN thin films. The decrease of binding energy indicates that with the increase of nitrogen content, the shielding of nuclear holes by electrons around ionized chromium atoms becomes less effective [32]. This also indicates that in these thin films, the charge neutralization around the Cr cation is weakened, and the charge neutralization around the N anion is enhanced. Therefore, the nitrogen content of T_130_ thin film is the highest, followed by T_82_ and T_44_ thin films, and T_26_ thin film is the lowest. The lattice constant increases with the increase of nitrogen content, which is consistent with the results of XRD. The higher the nitrogen content, the larger the lattice constant. With thickness increasing, the atomic diffusion is enhanced, which should also lead to more N content in the derived films [25,26,29]. The N 1*s*_1/2_ peak value of T_130_ thin film is centered at 396.25 eV, which is lower than that of chromium nitride with stoichiometry (396.7 eV) [2]. Such a low BE of N 1*s*_1/2_ implies that there is imbalanced stoichiometry and/or more absorbed N in the derived CrN films. Combined with the results of the XPS and XRD, it is certain that the thicker CrN films are at least close to in an even stoichiometric ratio.

**Figure 6:**
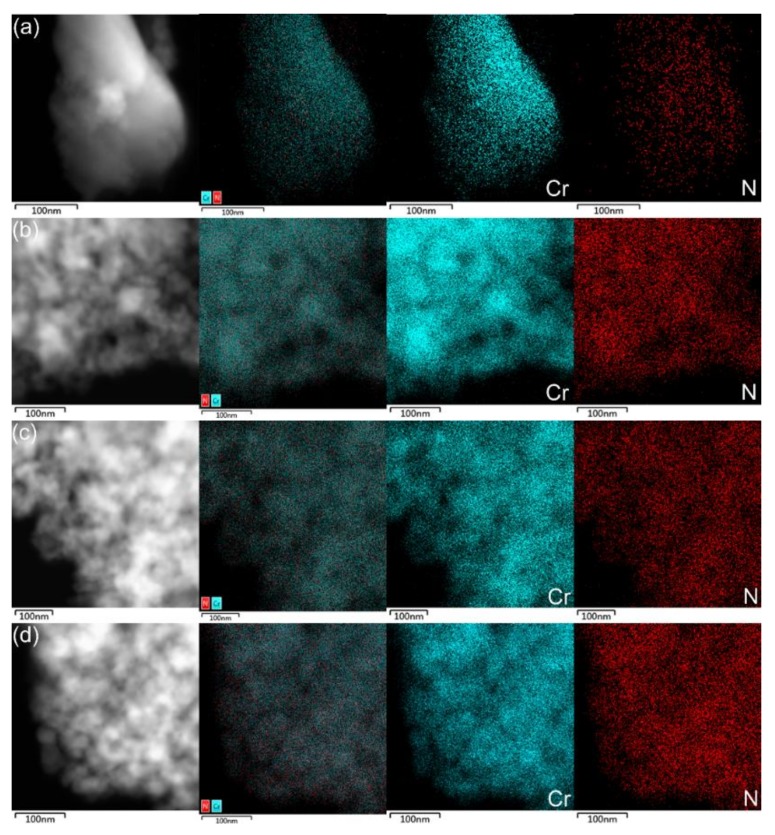
EDX elemental mapping measurements of the derived samples: (**a**) T_26_ thin film, (**b**) T_44_ thin film, (**c**) T_82_ thin film, (**d**) T_130_ thin film.

In order to further reveal the variation of nitrogen content and the stoichiometry of the thin CrN films with different thicknesses, energy-dispersive X-ray spectroscopy (EDX) elemental mapping measurements of the derived samples were carried out, and the results reveal the homogenous distribution of Cr and N, as shown in Figure 6. It is seen that as the thickness of the film increases, the intensity of the nitrogen element gradually increases, which is consistent with the XPS results. Since the detection depth of the EDX is the micrometre level, the distribution of the elements of all the samples is uniform. It was determined that the elemental composition of the inside film was consistent with that of the surface. Therefore, the results of the XPS can be sufficient to demonstrate the nitrogen content of the relevant whole films and the stoichiometric ratio of the elements. Additionally, as for the stoichiometry from EDX results, the accuracy for the light elements has a relatively high error, especially for the elements with atomic number less than 20 [33]. Therefore, it is necessary to point out here that N element is too light to be detected.

### 3.3. Electrical Transport Properties

**Figure 7:**
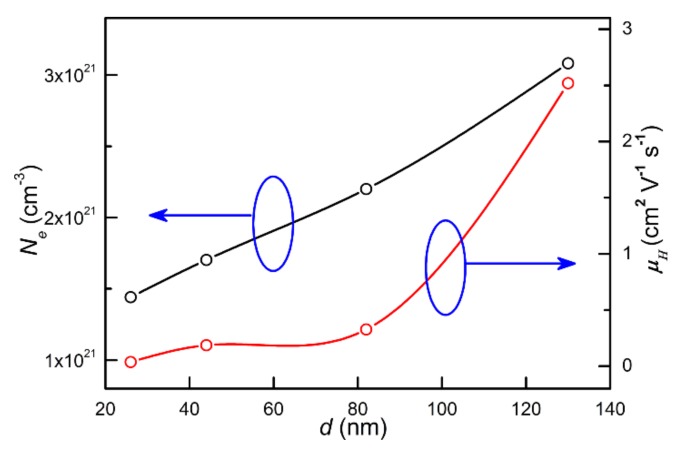
The electron carrier concentration and electron mobility dependence of the thickness for all derived CrN films.

In order to obtain the carrier concentration and mobility values of the CrN thin films with different thicknesses, the Hall measurements were performed at room temperature. From the results, the carrier type was found to be electronic-type (n-type), which is consistent with a previous report [6]. The carrier concentration *N_e_* and the mobility *μ_H_* were calculated to be 1.44 × 10^21^ cm^−3^ and 0.037 cm^2^V^−1^s^−1^ for the T_26_; 1.70 × 10^21^ cm^−3^ and 0.186 cm^2^V^−1^s^−1^ for the T_44_; 2.2 × 10^21^ cm^−3^ and 0.326 cm^2^V^−1^s^−1^ for the T_82_; and 3.08 × 10^21^ cm^−3^ and 2.52 cm^2^V^−1^s^−1^ for the T_130_, respectively, as shown in Figure 7. The corresponding values are listed in Table 2. The changes of carrier concentration and mobility are related to the variation of the nitrogen content, grain size and grain boundary scattering. From the analysis results, as the thin film thickness increases, the increasing nitrogen content and enlarged grain size increase the carrier concentration. At the same time, the reduction in the number of grain boundaries and scattering result in an increase of the mobility value. Consequently, the T_130_ thin film has the largest carrier concentration and mobility value, while the T_26_ thin film has the smallest corresponding value.

**Figure 8:**
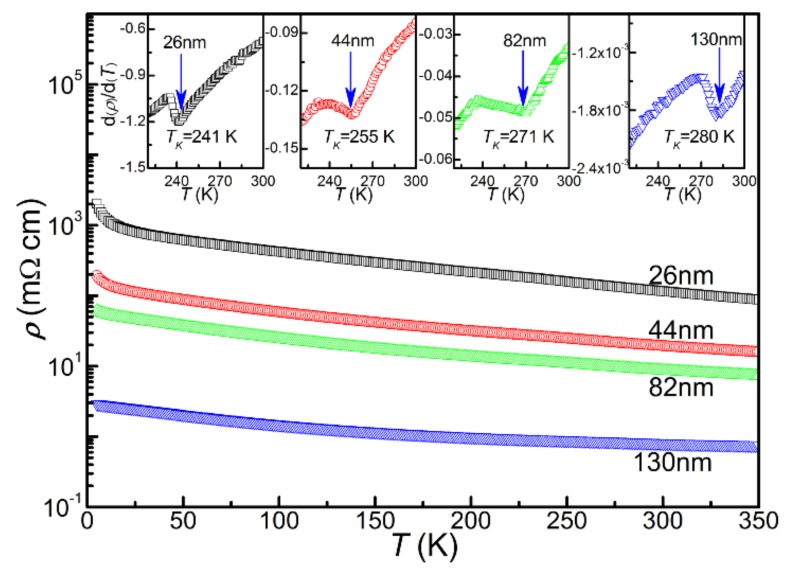
For the temperature dependence of resistivity for the derived CrN thin films, the d*ρ*/d*T* results to determine the *T_K_* are shown in the insets.

The temperature-dependent electrical resistivity of each of the derived CrN thin films under SrTiO_3_ substrates is shown in Figure 8 (current of 5 mA), and the obtained corresponding parameters are listed in Table 2. In order to obtain the influence of the substrate resistance on that of the thin film, we tried to measure the resistance of bare SrTiO_3_ substrate with 1000 °C post-annealing in the ammonia atmosphere. In fact, the resistance of the post annealed SrTiO_3_ substrate is very high, so it cannot be measured through four-probe method. Therefore, the relevant results of electrical transport measurements are intrinsic properties of the CrN thin films. In the temperature range of 5–350 K, all samples exhibited semiconductor-like behavior of d*ρ*/D*t* < 0. From the results, it can be seen that the resistivity decreases with the increase of thickness in the whole measuring temperature range. The resistivity values at 300 K (*ρ_300K_*) were 116.55, 19.28, 8.62 and 0.79 mΩ·cm for the T_26_, the T_44_, the T_82_ and the T_130_, respectively, which have been reported in the past for CrN ceramics and CrN thin films [6,7,8,34]. Combined with the microstructures and nitrogen content results, it is considered that the resistivity is primarily controlled by grain size and nitrogen content, which suggests that the enlarged grain size and the increased nitrogen content will reduce the value of the resistivity. Usually, at the Néel temperature, the orthorhombic Pnma structure of low temperature antiferromagnetism will change to the rock-salt Fm-3m structure of high temperature paramagnetism, showing a kink in the resistivity curve at the transition point [1,26]. The transformation temperature is defined as *T_K_*. As shown in the insets of Figure 8, the kink occurred at 241, 255, 271 and 280 K for the T_26_, T_44_, T_82_ and T_130_ respectively, as defined from the calculation results of d*ρ*/d*T*. Those results are similar to those of previous reports, and further confirm the thicker CrN thin films are at least nearly stoichiometric [6,35,36]. It is seen that the *T_K_* shows a large difference for the films with different thicknesses. According to the literature, *T_K_* is correlated closely with the nitrogen content in CrN [26]. From the XPS results, the binding energy values of the Cr 2*p*_3/2_ and the N 1*s*_1/2_ vary significantly from 576.47 to 574.71 eV and from 397.43 to 396.25 eV with increasing thickness. The obvious binding energy changes in Cr 2*p*_3/2_ and N 1*s*_1/2_ for the films with different thicknesses indicate the obvious changes in nitrogen content for the derived thin films, resulting in the obvious variation of *T_K_*.

**Figure 9:**
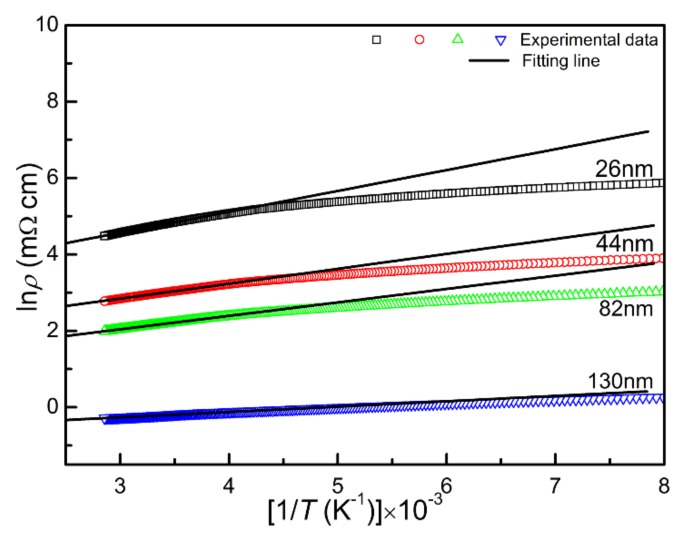
The electrical transport properties fitting results of the derived CrN thin films in the region above *T_K_*.

**Figure 10:**
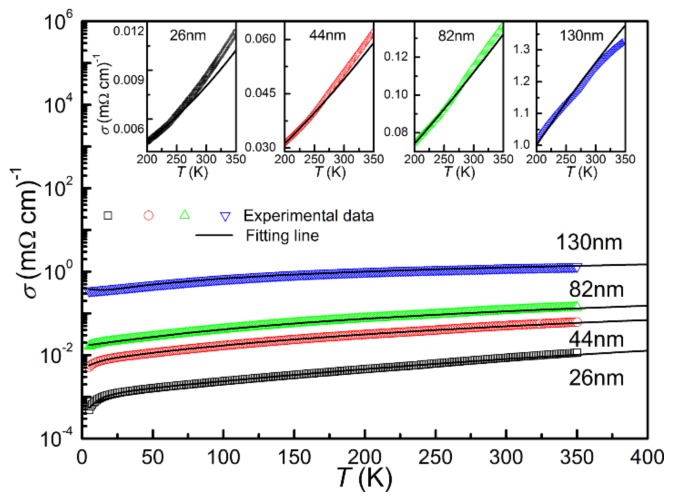
The electrical transport properties fitting results of the derived CrN thin films in the region below *T_K_*; the fitting results with high resolution are shown in the inset.

In order to study the electrical transport mechanism, the curve of resistivity versus temperature is fitted in different temperature ranges, as shown in Figure 9 and Figure 10. It is found that the resistivity at the temperature range above *T_K_* can be well fitted by the thermal activation model *ρ*(*T*) = *ρ_0_e*^−(*E*^*_g_*^/2*k*^*_B_^T^*^)^, a linear fit to ln*ρ* versus 1/*T* yields the band gap in the region above *T_K_*. The band gaps *E_g_* were 94.3, 67.3, 60.6 and 24.2 meV for the T_26_, T_44_, T_82_ and T_130_ respectively, which confirms the insulating state of the CrN thin films above *T_K_*, and those outcomes are the same as in a previous report [36]. The *E_g_* decreases with the increase of film thickness, which is related to the increase of nitrogen content and grain size.

As for the temperature range below *T_K_*, the electrical transport properties cannot be well fitted by thermal activation model. It was found that the resistivity can be well fitted considering the two-dimensional weak localization (2D-WL) model, as shown in Figure 10 [37,38]. The fitting results with high resolution are shown in the inset. This model based on the inelastic electron-electron and electron-phonon interactions is given by *σ*(*T*) = *σ*_0_ + A*T^p^* + Bln*T*. The second term of the equation accounts for the electron–electron or electron–phonon interactions [39]. Since the inelastic scattering increases with decreasing temperature, a natural logarithmic temperature dependent term is added to the equation [35,38]. For the value of the exponent *p* in the second term, if *p* = 0.5, the scattering process is dominated by electron–electron interactions for disordered systems [37]. However, if *p* > 0.5, the electron-phonon interactions dominate the scattering process. In this case, the electrical transport properties depend on the relative variation of phonon wavelength and the system dimension determined by the degree of disorder [39]. For the films with different thickness, the *σ*_0_ and *p* are 0.0004 mΩ^−1^cm^−1^ and 2.39 for the T_26_; 0.0017 mΩ^−1^cm^−1^ and 1.71 for the T_44_; 0.0122 mΩ^−1^cm^−1^ and 1.44 for the T_82_; and 0.3433 mΩ^−1^cm^−1^ and 1.11 for the T_130_, respectively. The values of *p* for all the derived thin films were greater than 0.5, indicating that the electron–phonon interactions dominate the scattering process. It is seen that the *p* decreases with thickness increasing, illustrating that the role of the phonon mediated scattering process weakens upon increasing thickness along with the electron-electron interactions [39].

### 3.4. The Zero-Field Sensitivity and Magnetoresistance Under High Magnetic Fields

**Figure 11:**
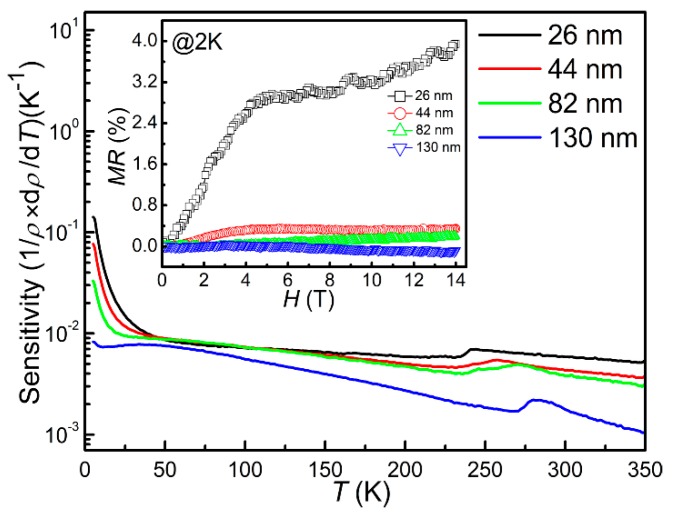
Zero-field sensitivity versus temperature of CrN thin film with different thickness. The magnetoresistance at 2 K is shown in the corresponding inset.

An important parameter for the cryogenic sensors is the zero-field sensitivity *S*. The larger the *S* value, the more sensitive to the temperature measurement of the sensors [13]. Figure 11 shows the temperature dependence of zero-field sensitivity of CrN films with different thicknesses in the temperature range of 5–350 K. The *S* values at 5 K were 0.14, 7.7 × 10^−2^, 3.3 × 10^−2^ and 8.3 × 10^−3^ K^−1^ for the T_26_, the T_44_, the T_82_ and the T_130_, respectively. The magnetoresistance *MR,* as another important parameter, is required to be as low as possible [10,11]. The inset of Figure 11 shows the *MR* results at 2 K of the derived CrN thin films. The *MR* values at 2 K under 14 T were 3.94%, 0.34%, 0.20% and −0.10% for the T_26_, the T_44_, the T_82_ and the T_130_, respectively. As can be seen from the illustration, the MR values of T_130_ thin films are negative, which is as it was the previous report [40]. It is due to the enhancement of antiferromagnetic spin alignment and the reduction of carrier scattering by applying magnetic field. With thickness increasing, the disorders in the CrN thin films are decreased due to the improved stoichiometric ratio and the enlarged grain size, resulting in the smaller *MR* value as thickness increases. The results show that the CrN thin films have the high sensitivity, larger than 10^−2^ K^−1^ at 5 K, and low magnetoresistance, less than 1%, at 2 K under 14 T, which is comparable to the commercial sensors CERNOX in previous reports [10,11,12,13,41].

## 4. Conclusions

In summary, CrN thin films with different thicknesses were fabricated by chemical solution deposition on SrTiO_3_ single crystal substrates. Thickness’s effects on microstructures and electrical transport properties were studied. It was observed that all the derived CrN thin films showed a semiconductor-like electrical transport behavior. Increasing the thickness resulted in an increase in the grain size and nitrogen content. The resistivity, zero-field sensitivity and magnetoresistance are decreased upon increasing the thickness of CrN thin films. The obtained large zero-field sensitivity can be effectively tuned to 10^−2^ K^−1^ at 5 K with the ultra-low magnetoresistance less than 1% at 2 K under 14 T by reasonably controlling the thickness, suggesting CrN thin films can be considered a new type of candidates for cryogenic sensor under high magnetic fields. The research results in this paper expand the application range of chemical solution method, and demonstrate extensive basic research on the physical properties of thin films, which provides an effective method for preparing other new novel nitride thin films.

## Figures and Tables

**Table 1:** The microstructure and binding energy parameters of CrN thin films. (Lattice constant of the CrN thin film *a_f_*).

CrN	*a_f_* (Å)	Average Grain Size (nm)	Binding Energy of Cr 2*p*_3/2_ (eV)	Binding Energy of N 1*s*_1/2_ (eV)
T_26_	4.140	26	576.47	397.43
T_44_	4.143	31	575.65	397.11
T_82_	4.145	44	575.51	396.89
T_130_	4.148	73	574.71	396.25

**Table 2:** The electrical transport and magnetoresistance parameters of CrN thin films. (Carrier concentration *N_e_*, mobility *μ_H_*, resistivity at 300 K *ρ*_300K_, transformation temperature *T_K_*, zero-field sensitivity at 5K *S_5K_*, magnetoresistance *MR*).

CrN	*N_e_* (cm^−3^)	*μ_H_* (cm^2^V^−1^s^−1^)	*ρ*_300K_ (mΩ·cm)	*T_K_* (K)	*S_5K_* (K^−1^)	*MR* at 2K Under 14 T (%)
T_26_	1.44 × 10^21^	0.037	116.55	241	0.14	3.94
T_44_	1.70 × 10^21^	0.186	19.28	255	7.7 × 10^−2^	0.34
T_82_	2.20 × 10^21^	0.326	8.62	271	3.3 × 10^−2^	0.20
T_130_	3.08 × 10^21^	2.520	0.79	280	8.3 × 10^−3^	−0.10
